# Phase Equilibria of the In–Pd–Sn System at 500 °C and 800 °C: Experimental Study and CALPHAD Modeling

**DOI:** 10.3390/ma16041690

**Published:** 2023-02-17

**Authors:** Alexandr S. Pavlenko, Elizaveta G. Kabanova, Maria A. Kareva, Evgeniya A. Ptashkina, Alexander L. Kustov, Galina P. Zhmurko, Victor N. Kuznetsov

**Affiliations:** 1 Department of Chemistry, Lomonosov Moscow State University, Moscow 119991, Russia; 2 Institute of Ecotechnologies, National University of Science and Technology MISiS, Leninsky Prospect 4, Moscow 119991, Russia

**Keywords:** In–Pd–Sn system, ternary phase diagram, isothermal sections, thermodynamic modeling

## Abstract

Phase equilibria in the In–Pd–Sn system were investigated by a combination of key experiments and thermodynamic modeling. Partial isothermal sections at 500 °C and 800 °C of the In–Pd–Sn system for Pd contents above 66 at.% have been plotted experimentally using scanning electron microscopy with energy-dispersive X-ray spectroscopy (SEM/EDX) and X-ray diffraction (XRD). The solubility of the third component in binary compounds InPd_3_ and Pd_3_Sn was determined. The new ternary compound τ_1_ was found in Pd contents ranging from 20 to 25 at.% and at Sn contents varying from 5 to approximately 17 at.% Sn. This compound crystallizes in an Al_3_Ti-type tetragonal structure. Isostructural InPd_2_ and Pd_2_Sn phases from the In–Pd and Pd–Sn binary compositions form a continuous phase field in the ternary system at both temperatures. The temperatures of the solidus, liquidus, and phase transitions of the alloys along the Pd–In50Sn50 line were measured using DTA/DSC. Thermodynamic calculation of the In–Pd–Sn ternary system is performed using the CALPHAD method using the Thermo-Calc^®^ software. The thermodynamic properties of the disordered fcc and liquid phases were described by the Redlich–Kister–Muggianu model. To describe intermetallic phases, namely, InPd_3_, Pd_3_Sn, τ_1_ and Pd_2_(In_x_Sn_1−x_), a two-sublattice models was used. Thermodynamic description of the In–Pd–Sn system obtained in this study is in good agreement both with our results and the published experimental data

## 1. Introduction

Palladium-based alloys are widely used in chemical, microelectronics, medicine, and a number of other industries. Components of such alloys are often low-melting non-transition metals, which improve casting characteristics and may serve as strengtheners [1]. They also form numerous intermetallic compounds with palladium. These compounds have a few positive properties, for example, they can be used as highly efficient, selective, and stable catalysts [2,3]. On the other hand, their formation can cause undesirable effects. For example, when using lead-free solders containing indium and tin, the formation of intermetallics can seriously deteriorate the mechanical and conductive properties of the solder joints in printed circuit boards [4,5]. In both cases, information on the conditions of formation, stability, and structure of intermetallic compounds is required, which is obtained in studies of palladium and low-melting metals phase diagrams. 

However, experimental studies of phase equilibria in multi-component systems in a wide range of temperatures and compositions is a time- and labor-consuming process. Here, thermodynamic modelling (CALPHAD) may be of great use because it allows for obtaining thermodynamic description of the system and anticipating phase equilibria and phase properties in uninvestigated areas of the diagram based on a limited experiment. Moreover, with binary and ternary border system descriptions, it allows forecasting phase equilibria in systems composed of four or more components. This is why obtaining the thermodynamic description for the In–Pd–Sn ternary system is an actual task.

A part of the In–Pd–Sn system phase diagram with a palladium content below 60at.% was studied previously [6]. The authors used X-ray diffraction (XRD), energy-dispersive X-ray spectroscopy, and scanning electron microscopy (SEM/EDX) Based on the study results, three isothermal sections at 200 °C, 500 °C, and 700 °C were obtained. It was found that the third component solubility in InPd, In_3_Pd_2_, In_7_Pd_3_, PdSn, Pd_20_Sn_13_ phases at 500 °C does not exceed ~7 at.%. The phase based on the Pd_2_Sn compound exists in the ternary along the palladium iso-concentration line up to ~22 at.% In. The tin solubility in InPd and that of indium in Pd_20_Sn_13_ at 700 °C increased (crystal structures of binary compounds in the In–Pd and Pd–Sn systems are presented in Table 1). The authors found no ternary compounds in the area of the compositions studied [6].

The authors [12] studied thermodynamic properties of the In–Pd–Sn system. Using the drop calorimetric technique, partial and integral mixing enthalpies were determined for liquid alloys at 900 °C with up to 40 at.% palladium content.

For thermodynamic modeling of the ternary system, reliable descriptions of binary limiting systems are necessary. In this study, critical analysis of the existing thermodynamic descriptions in the In–Pd and Pd–Sn system was performed. Special attention was paid not just to experimental results reproducibility, but to mutual consistency of models of isostructural phases and of Gibbs energies of pure components in all the systems. Based on that, the authors made the following conclusions:

- The thermodynamic description of the In–Pd system [13] uses the up-to-date Gibbs energies of components [14] and reproduces well the results of experimental studies of phase equilibria and phase thermodynamic properties. This description can be accepted in this work without any changes.
- The thermodynamic calculation of the In–Sn system was performed in three works [15,16,17]. The results in [15] are in the best agreement with the experimental data for phase equilibria. According to other studies, the γ phase present in the system turns out to be too stable, which is why its calculated homogeneity field is much wider than the experimental one. In all the In–Sn system calculations [15,16,17], obsolete values [18] of indium stability parameters in the β tin-type structure and of tin in the In type structure were used. The description of the In–Sn system requires revision.
- The most complete thermodynamic description of the Pd–Sn system, taking into account the latest studies of phase equilibria in the system, was proposed in [19]. Two models for the liquid phase: the Redlich–Kister polynomial and the associated solution theory were used. Both provide essentially the same quality of the reproduction of all the experimental data available.

Thus, for thermodynamic modeling of the In–Pd–Sn ternary, additional experimental data are required after studying the phase equilibria in this system at palladium content above 60 at.%. In addition, the thermodynamic description of the In–Sn binary system should be revised.

## 2. Experimental Procedures

In total, 32 alloys weighing 1 g each were obtained to study phase equilibria in the In–Pd–Sn system. Powder palladium (99.95% wt.), tin wire (99.95% wt.), and indium bars (99.999% wt.) were used as source materials. The alloys were produced in a Buehler MAM 1 arc furnace in an ultra-pure argon (99.9999%) atmosphere purified by preliminary melting of a getter (hafnium). Afterwards, the samples were annealed in evacuated silica ampoules with subsequent quenching in cold water. The annealing times were chosen according to our experience with the similar systems. At 800 °C, the annealing time was 1680 h (14 samples), and at 500 °C for 3600 h (18 samples). The high content of palladium in the alloys and the previous studies of the authors of similar systems with palladium [20,21] were taken into account when choosing the annealing time. 

The alloys obtained were examined by X-ray diffraction (XRD), scanning electron microscopy (SEM), energy-dispersive X-ray (EDX) analyses, and differential thermal analysis (DTA). XRD was performed by a DRON-4 diffractometer using monochromatized CuKα radiation (graphite monochromator in the secondary beam) and by STOE STADI P diffractometer, using monochromatized CuKα1 radiation (germanium monochromator). The obtained sets of reflections were indexed using the STOE Win XPOW (Ver. 2.24) software package.

Metallographic studies and EDX analysis were performed using Carl Zeiss LEO EVO 50XPV scanning electron microscope with an INCA Energy 450 energy dispersive analysis system (Oxford Instruments) operating at an accelerating voltage of 30 kV and a beam current of 30 μA. A Q-BSD detector was used to increase the image contrast. To determine the composition of the phase, the content of each element was determined at 3–5 randomly selected points belonging to the phase. The averages of the phase composition were taken from all the points. The composition of the alloy was determined in a similar way. The content of each component was measured in three randomly selected areas of the sample surface. The overall composition was taken as the average over three values.

The liquidus and solidus temperatures were determined by DTA, using STA 449 F1 Jupiter thermal analyzer (Netzsch, Germany). Runs were performed with a scanning rate 20 °C min^−1^. 

## 3. Thermodynamic Model

The thermodynamic description of In–Pd–Sn system was obtained following the CALPHAD approach. Here, each phase is assigned its own model according to the structure, composition and other factors. The Gibbs energy function of temperature and composition contains some parameters to be evaluated [22].

Thermo-Calc^®^ software (version 2021b) was used for calculations. The Gibbs energy functions of the phases of the pure components were extracted from PURE5 database provided with the Thermo-Calc^®^ software package [14]. 

The liquid (L) and fcc (α) disordered phases were described by the substitutional solution model where the Gibbs free energy $G^{\psi }$ is given as:

(1) Gψ(xi,T)=∑ixi°Giψ(T)+RT∑ixiln(xi)+GXsψ(xi,T)

where $x_{i}$ is mole fraction of a component *i*, and $G_{i}^{\psi }$ is Gibbs energy of the component *i* in the structure of Ψ phase.

The excess Gibbs energy term GXsψ in (1) were modelled by the Redlich-Kister polynomial [22,23]:

(2) G Xs(xi, xj)=xixj∑v=0nL vi,j(xi−xj)v

Here, *L_i,j_* coefficients are interaction parameters that are generally to be assessed. 

### Thermodynamic Models of Phases with Sublattices 

To describe intermetallic phases, the conventional sublattice model [22] was taken. Here, the Gibbs energy of a phase with sublattices is expressed as:

(3) Gψ(xi,T)=∑ (∏ yisΔf0Gend)+RT∑s∑iasyislnyis+GXsψ

where $\Delta_{f}^{0}G_{end}$ is the Gibbs energy of the endmembers, $a^{s}$ is the stoichiometric coefficient of *s* sublattice, $y_{i}^{s}$ is the site fraction of *i* species in s sublattice. The excess Gibbs free energy GXsψ  includes the interaction parameters of the components in the sublattices, which can be optimized.

The parameters of the phase models were evaluated in two steps. Reasonable initial values were estimated by trial and error. Where possible, the values were refined through the PARROT module of the Thermo-Calc^®^ software (version 2022b).

## 4. Results and Discussion

### 4.1. Experimental Investigations

The results of XRD and EDX analyses of the samples at 500 and 800 °C are listed in Table 2 and Table 3. 

**Figure 1:**
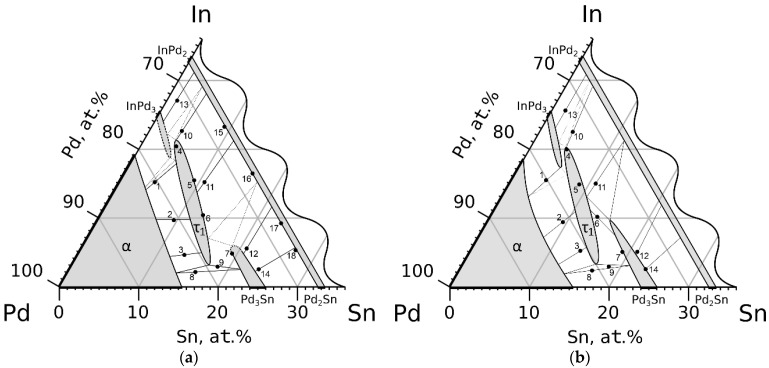
The In–Pd–Sn system isothermal sections at 500 °C (**a**) and 800 °C (**b**) with sample numbers.

Palladium-rich areas of isothermal sections of the In–Pd–Sn system at 500 °C and 800 °C, built according to the data from Table 2 and Table 3, are shown in Figure 1. Both are very similar, with only small differences in the solubility of the third components in InPd_3_ and Pd_3_Sn compounds. 

At both temperatures, the α-solid solution boundary is determined basing on the results of the study of samples 1–3, 8 and 9 (Table 1 and Table 2). It almost coincides with the line connecting the values of indium and tin solubility in palladium. Just as in both boundary systems, it almost does not change with the temperature. 

The existence of continuous solubility between the phases InPd_2_ and Pd_2_Sn with orthorhombic structure of the Co_2_Si type at 500 °C was confirmed by studies of samples 10–18, whereas at 800 °C, by the studies of samples 10–14. The compositions of single-phase samples and tie-lines of two-phase equilibria at two temperatures are plotted on the isothermal triangles (Figure 1) according to EDX data (Table 2 and Table 3). 

Three phases are in equilibrium with the α-solid solution and the Pd_2_(In_x_Sn_1−x_) phase in the In–Pd–Sn system: a low-temperature modification of the InPd_3_ compound with the Al_3_Zr-type crystal structure, τ_1_ ternary phase with Al_3_Ti-type structure, and Pd_3_Sn-based solid solution with the AuCu_3_-type structure. Note that all the above structure types are derived from the Cu-type structure by ordering (AuCu_3_) or ordering with simultaneous tetragonal lattice distortion (Al_3_Zr, Al_3_Ti). 

Note that the crystal structures of both the InPd_3_ and τ_1_ phases as identified from the powder patterns appearing as disordered In-type structures. Since the atoms of indium, tin, and palladium have very similar values of atomic X-ray scattering factors [24], superstructure reflections intensity was essentially zero. For this reason, to determine the crystal structure of In-type tetragonal phases in samples 1–6, 8–11, and 13, we used an approach similar to that described in [20,21]. This approach is based on the results of [8,9,25], who found that in the In–Pd binary system, the low-temperature modification of InPd_3_ (Al_3_Ti-structure) and the high-temperature modification of the InPd_3_ compound (Al_3_Zr-structure) differ in the ratio of c/a parameters for their face-centered pseudocubic subcells. The c/a ratio equal to 0.935 corresponded to the Al_3_Zr-type structure, and the c/a equal to 0.91 corresponded to the Al_3_Ti-type structure. 

**Figure 2:**
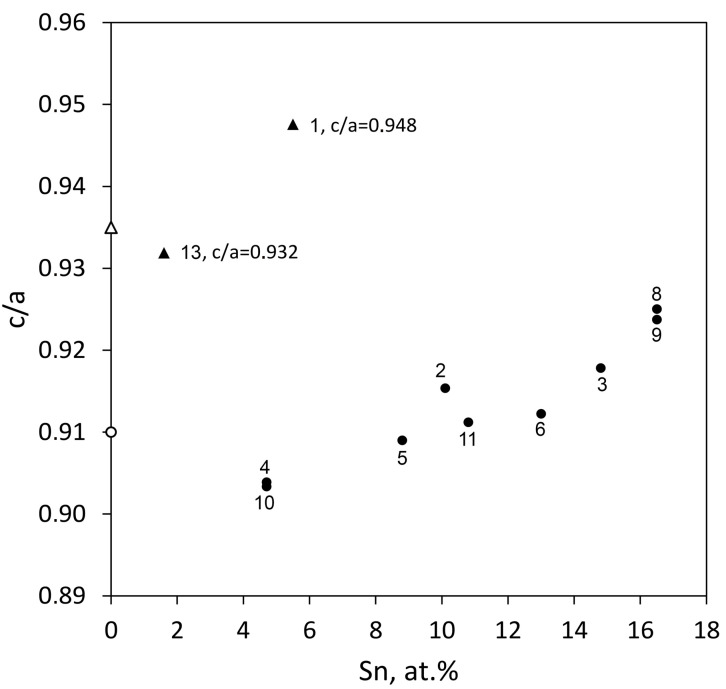
Dependence of the c/a values of pseudo-cubic sub cell of InPd_3_ (triangles) and τ_1_ (circles) phases on the tin content (samples annealed at 800 °C).

**Figure 3:**
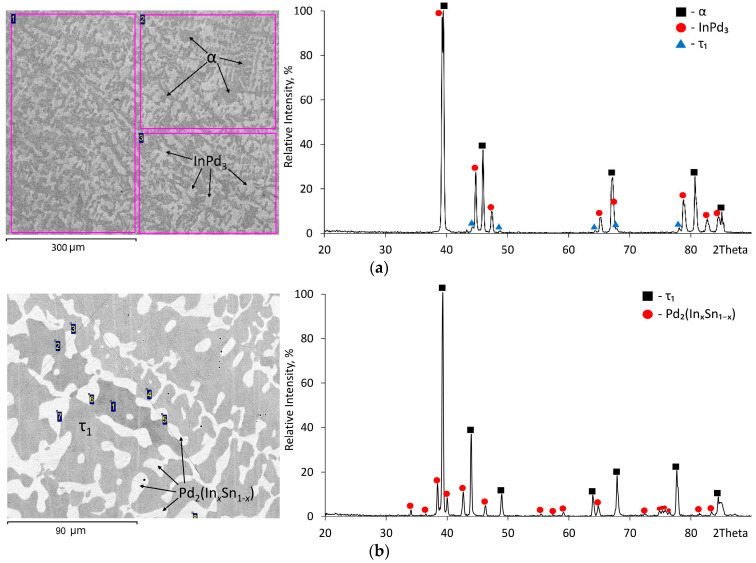

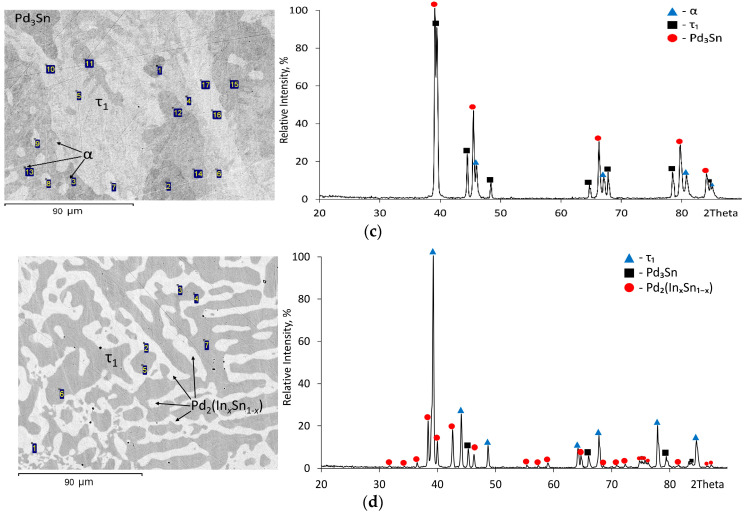
BSE images and XRD patterns of the In–Pd–Sn alloys annealed at 800 °C: (**a**) No. 1, (**b**) No. 10, (**c**) No. 9, (**d**) No. 11; the numbers indicate the points of measurement of the composition.

Figure 2 shows the dependence of the c/a values of tetragonal phases in Samples 1–6, 8–11 and 13 annealed at 800 °C on tin content. In the samples with a tin content of up to 5.5 at.% (samples No. 13 and 1), the c/a ratio varies from 0.93 to 0.945, and the remainder from 0.905 to 0.92. This indicates that at 800 °C and a content of Sn of up to ~6%, the low-temperature modification of InPd_3_ (Al_3_Zr-structure) exists, whereas at a content of Sn from ~5 to ~17 at.%, the τ_1_ phase with the Al_3_Ti-type structure is stable. The minimum tin content in the τ_1_ phase was determined by the study of sample 10, and the maximum one is determined by the composition of the τ_1_ phase in the three-phased sample 9. BSE images and XRD patterns of Samples 1, 10, and 9 annealed at 800 °C are presented in Figure 3.

The XRD data of Sample 1 at 800 °C (Table 2, Figure 3a) indicate the presence of an α-solid solution and two tetragonal phases with c/a values of 0.948 and 0.915. As noted above, the first one refers to the InPd_3_ compound, the second one refers to the τ_1_ phase. The EDX analysis did not determine the composition of the τ_1_ phase due to its very low content in the sample; however, the α phase (In12.7Pd83.7Sn3.6) and InPd_3_ (In17.0Pd77.5Sn5.5) uniquely determine one side of the tie triangle α + InPd_3_ + τ_1_, as well as the tin solubility (~6 at.%) in the InPd_3_ compound at 800 °C. On the contrary, the results of EDX and XRD for that sample annealed at 500 °C show a τ_1_ phase composition with somewhat lower (~5 at.%) tin content. This suggests that when the temperature drops from 800 °C to 500 °C, tin solubility in InPd_3_ decreases insignificantly. 

**Figure 4:**
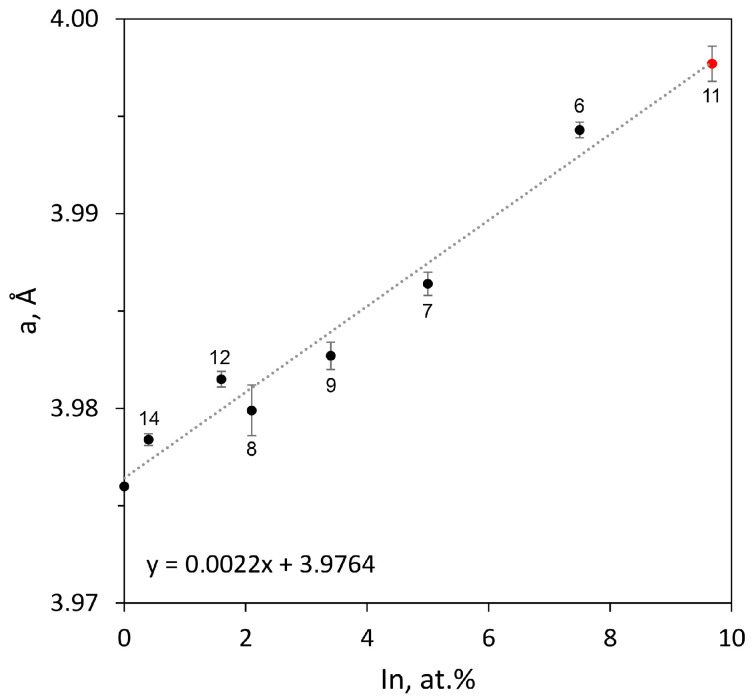
Dependence of the lattice parameter of the Pd_3_Sn phase on the indium content.

The homogeneity field of the Pd_3_Sn phase at 800 °C was determined by the XRD method for the two-phased samples 6, 12, and 14 and the three-phased samples 8, 9, and 11 (Table 2). The maximum solubility of indium in the Pd_3_Sn phase was determined in the study of Sample 11 s. According to the XRD, the sample relates to the three-phase equilibrium of Pd_2_(In_x_Sn_1−x_) + Pd_3_Sn + τ_1_ (Figure 3d). However, EDX was unable to determine the composition of Pd_3_Sn. Therefore, the solubility of indium in the Pd_3_Sn phase was found from dependence of the lattice spacing of that phase on In content (Figure 4). It is close to ∼10 at.%. 

At 500 °C, the solubility of indium in Pd_3_Sn is less than 10 at.% since at such an indium content, not Pd_3_Sn, but the τ_1_ phase of (In10.4Pd76.3Sn13.3) composition is formed (Table 3, sample 6). Thus, the solubility of the third component in the Pd_3_Sn phase decreases to some extent, as in the InPd_3_ phase. 

### 4.2. Differential Thermal Analysis

The results of the studies of isothermal sections were supplemented with experimental data on temperatures of phase transitions of four alloys obtained using DTA/DSC methods. Four samples annealed at 500 °C with compositions on a line connecting pure palladium and the equiatomic composition of the In–Sn system were selected. Sample 19 from the two-phase region Pd_2_(In_x_Sn_1−x_) + Pd_20_Sn_13_ was synthesized. The results of the DSC study are presented in Table 4. 

**Figure 5:**
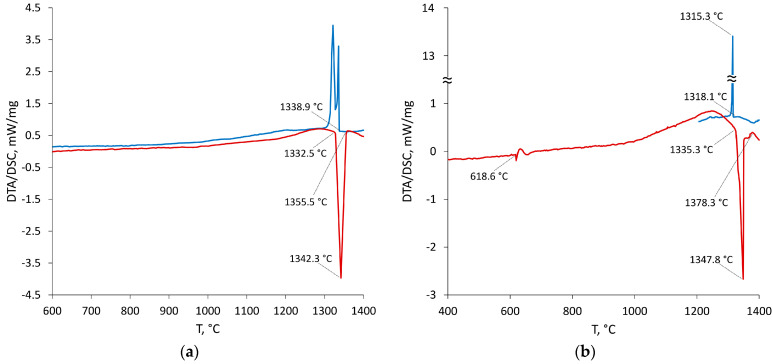
Heat curves from Samples 2 (**a**) and 6 (**b**). Red line—heating; blue line—cooling.

The solidus temperature was determined as the onset of the peak of melting on the heating curve. Obvious supercooling was observed during crystallization, so the liquidus temperature was determined as the last peak on the heating curve (Figure 5). The temperatures of all phase transitions on the samples studied are resumed in Table 4. The liquidus and solidus temperatures were used in determining of phase models’ parameters, whereas temperatures of other phase transitions were used for validation of calculations. 

Heat curves for Samples 2 and 6 are shown in Figure 5a,b. 

Sample 2 started to melt at 1333 °C and ended at 1342 °C. In Sample 6, the heat curve showed that the effect at 619 °C corresponds to the τ_1_→(τ_1_ + Pd_3_Sn) transition. This is confirmed by the fact that Sample 6 annealed at 800 °C contains the Pd_3_Sn compound in addition to the τ_1_ phase (Table 2). The melting starts at ∼1335 °C and ends at 1378 °C.

### 4.3. Thermodynamic Modelling

#### 4.3.1. In–Sn System

As noted above, the thermodynamic calculation of the In–Sn system [15] is in good accordance with the experimental data on phase equilibria and phase thermodynamic properties. However, it used outdated Gibbs energy values for the tin in the In-type structure (TET_ALPHA1) and for indium in the β-Sn type structure (BCT_A5), so the available calculation for the In–Sn system had to be revised in this work. Taking into account the new values of stability parameters, $G_{Sn}^{TET\_ALPHA1}$ and $G_{In}^{BCT\_A5}$, a good agreement with the experiment was achieved by changing the parameters of the indium–tin interaction in these two phases, and the parameters of models of other phases were left unchanged. Optimization of the parameters of the β-phase and tin-based solid solution was performed using the PARROT module of Thermo-Calc software using as input experimental data on phase boundaries and coordinates of invariant equilibria [26,27,28,29].

**Figure 6:**
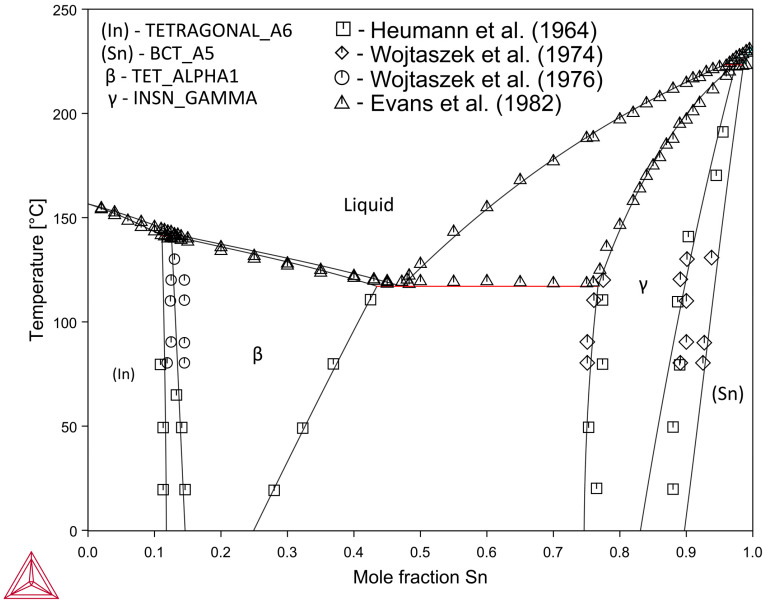
Calculated phase diagram of the In–Sn system along with the experimental data [26,27,28,29].

Figure 6 shows the In–Sn system equilibrium diagram calculated using the parameters obtained.

#### 4.3.2. In–Pd System

Since the ternary phase τ_1_ found in the In–Pd–Sn is isostructural with the βInPd_3_ phase of the In–Pd binary, we assumed that the addition of tin stabilizes the high-temperature modification of the InPd_3_ compound, and the single model was used for both phases. For the β-InPd_3_ phase [13] suggested a formal two-sublattice model (Pd)_0.74_(In)_0.26_ which corresponds to the phase composition. However, in the ternary, palladium content in τ_1_ phase varies from 74 at.% to 80 at.%. To account for these data, the β-InPd_3_ phase model was expanded to (Pd)_0.74_(In,Pd)_0.26._ The parameters $G_{In:Pd}^{}$ and ^0^$L_{In,Pd:Pd}^{}$ were determined by optimization, which used the data for both In–Pd and In–Pd–Sn systems simultaneously. The parameters of models of all other phases of the In–Pd system remain unchanged. As a result, we managed to preserve and, in some cases, even improve the description of temperatures and compositions of invariant equilibria with participation of this phase in the In–Pd binary (Table 5). The resulting parameters of the βInPd_3_ phase model are listed in Table 6.

#### 4.3.3. Pd–Sn System

For the Pd_2_Sn phase of the Pd–Sn system, a single-sublattice model (Pd_2_Sn)_1_ was used [19], whereas a two-sublattice model (Pd)_0.667_(In)_0.333_ was proposed in [13] for the InPd_2_ phase of the In–Pd system. As these phases actually form continuous solid solution in the ternary, similar models should describe both. For this purpose, the model of the Pd_2_Sn phase in the Pd–Sn system was replaced by the (Pd)_0.667_(Sn)_0.333_ one. The stability parameter of the single end-member Pd_2_Sn was determined as G(Pd:Sn)Pd2Sn=13GPd2SnPd2Sn. 

The above-mentioned model of the βPd_3_In/τ_1_ phase in the ternary transformation turns into the Pd_0.74_(In,Pd,Sn)_0.26_ one. The Pd:Pd and Pd:In endmembers as well as the Pd:(In,Pd) interaction corresponding to the In–Pd binary. Their values were determined during the revision of its description. 

The values of the Gibbs energy function of the Pd:Sn endmember and the interaction parameter Pd:(Pd,Sn) correspond to the virtual phase in the Pd–Sn edge. The phase with gross composition Pd_0.8_Sn_0.2_ was found as metastable still in 1957 [31]. In the ternary, the τ_1_ phase closely approaches that composition at both 500 and 800 °C. Moreover, no significant slope changes between the fcc/fcc + τ_1_ and fcc/fcc + Pd_3_Sn boundaries were detected in the present work. This means that in the Pd–Sn binary, the stability (Gibbs energy) of the virtual τ_1_ phase with the Al_3_Ti-type structure is close to the stability of the phase with a Cu_3_Au-type structure. 

When the stable phase Pd_3_Sn is suspended for some reason, the “nearly stable” τ_1_ phase should be in equilibrium with the fcc solid solution and the Pd_2_Sn compound. The coordinates of these metastable equilibria were estimated by extrapolation of experimental ternary equilibria towards the Pd–Sn side. 

The difference between enthalpies of formation of Pd_3_Sn phases with Cu_3_Au and Al_3_Ti type structures was taken from the ab initio calculation presented in the OQMD [32]. Gibbs energy function of the virtual Pd:Sn end-member of the τ_1_ phase was calculated by adding of this difference to the Gibbs energy function of stable Pd_3_Sn phase [19] Other parameters of the virtual τ_1_ phase in the Pd–Sn binary were obtained from estimated metastable equilibria with its participation.

#### 4.3.4. In–Pd–Sn Ternary System Modeling

The initial data for calculating the In–Pd–Sn ternary system were the enthalpy of mixing of liquid alloys [12], data on phase equilibria obtained in the present work and taken from [6], and the phase transition temperatures obtained in the present work. The optimization also used the liquidus temperatures determined by kinks (slope breaks) of dependence of mixing enthalpy on concentration at 900 °C [12].

The parameters of the model of phases in the In–Pd–Sn system were determined as follows. First, the parameters of the phase models of all the phases were determined basing on phase equilibria separately at each temperature of 500 °C, 700 °C, 800 °C, and 900 °C. The resulting values were approximated using the *a* + *b*·*T* function. After that, all parameters were jointly optimized in the PARROT module of the Thermo-Calc^®^ software package, taking into account all the above experimental data.

For each temperature, the parameters of the ternary interactions for α phase were determined, without which the stability of the phase would be too high, which, in turn, would significantly change the character of the calculated phase equilibria.

The next phase was the liquid. The authors of [12] noted that their experimental data on the enthalpies of mixing of liquid alloys are well described by the Toop model. However, its software support, for example in Thermo-Calc^®^, is much weaker than that of more commonly used Muggianu model. Moreover, the latter was used in our previous calculations of related systems (f.e. [21]), and unification of models is highly desirable. In addition, the present authors tested [33] the performance of Toop and Muggianu models using the Ag–Au–In system as test case. With inclusion of proper ternary interactions both provided the results of nearly the same quality. For these reasons the parameters of the ternary interaction, in the first approximation, were chosen as follows. The excess Gibbs energies of melt were calculated in a dense grid of temperatures and compositions using Toop’s model. Then, the resulting values were approximated using Muggianu’s model with the ternary interaction parameters. The parameters, approximating the results of Toop calculation, were slightly changed in subsequent optimization.

After obtaining the model parameters for the liquid and α phase, other phases were added sequentially: In_3_Pd, τ_1_, Pd_3_Sn, In_7_Pd_3_, PdSn_2_, Pd_2_(In_x_Sn_1−x_), Sn_20_In_13_, InPd and PdSn.

The resulting parameter values are presented in Table 6. Those called “estimated” were fixed on the values obtained at the first stage of calculations. 

**Figure 7:**
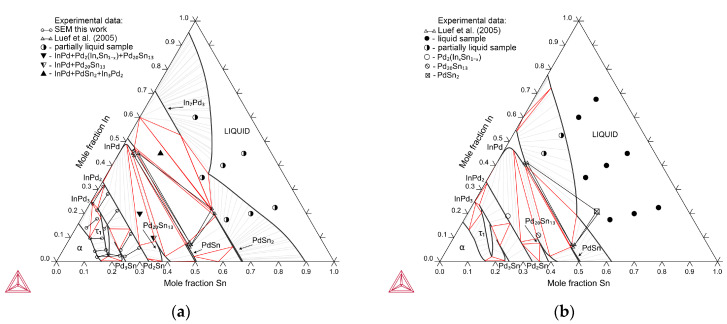

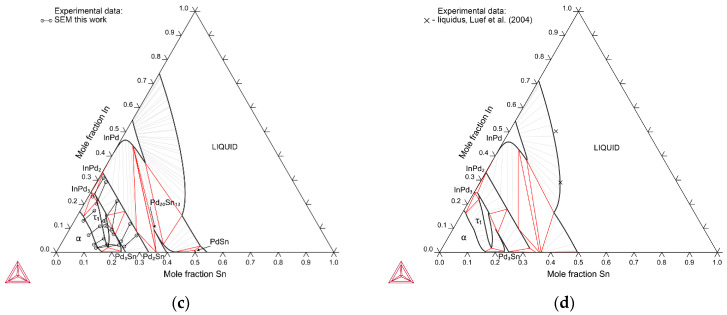
The In–Pd–Sn system calculated isothermal sections at 500 °C (**a**), 700 °C (**b**), 800 °C (**c**), and 900 °C (**d**) [6,12].

The calculated isothermal sections of the In–Pd–Sn ternary are shown in Figure 7. Good agreement between the calculation results using experimental data on phase equilibria [6] and the results of this study can be noted. 

It should be noted that a three-phase triangle (InPd + PdSn + PdSn_2_) is present on the isothermal section at 700 °C. However, the PdSn_2_ phase in the Pd–Sn system is formed only at 600 °C. As noted by the authors [6], the three-phase samples were partially melted. It is likely that the composition attributed to the PdSn_2_ phase actually corresponds to a crystallized melt.

**Figure 8:**
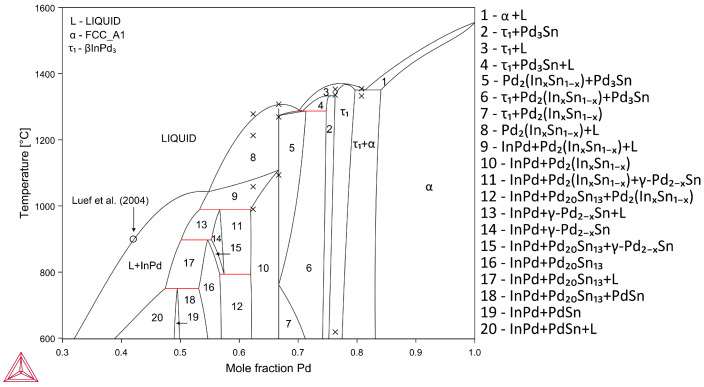
Pd–In50Sn50 part of the In–Pd system polythermal section in comparison with the DTA results (crosses indicate DTA results; circles indicate data [12]).

Figure 8 shows a comparison of the calculated and experimental polythermal section Pd–In_50_Sn_50_ of the In–Pd–Sn ternary. Experimental data are presented as transition temperatures obtained in this work. Note that only the liquidus and solidus temperatures were used for the search of parameters of phase models. The good agreement between the calculated and experimental temperatures of other phase transitions confirms that the obtained description of the In–Pd–Sn ternary system is correct.

**Figure 9:**
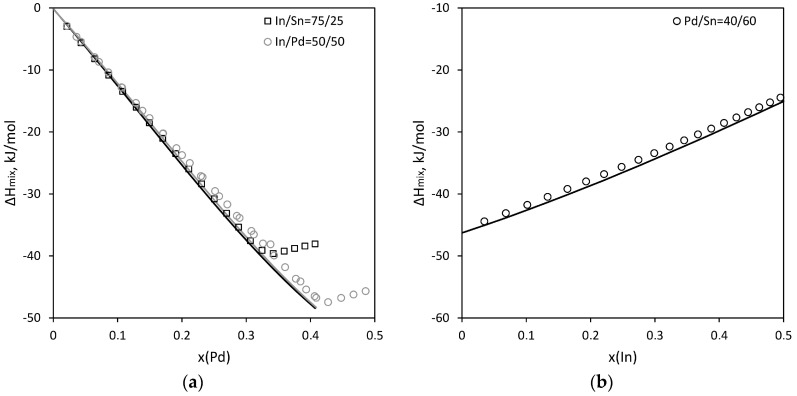
The enthalpy of formation of the melt for sections (**a**) Pd–In75Sn25 and Pd–In50Sn50; (**b**) In–Pd40Sn60. Dots indicate experimental data [12].

Figure 9 shows a comparison between the calculated and experimental mixing enthalpy models for the liquid phase [12]. It can be noted that there is an excellent agreement between them. 

## 5. Conclusions

Isothermal sections in the palladium-rich region of the In–Pd–Sn ternary system at 500 °C and 800 °C were determined using SEM, EDX and XRD methods. A ternary compound τ_1_ isostructural with a high-temperature modification of the βInPd_3_ phase was found. The ternary compound exists in the range of 4 at% to 16.5 at% (at 500 °C) or 18 at.% Sn (at 800 °C). At the same time, the palladium content in this phase increases from ~75 at.% to 80 at.%. Phase transition and melting temperatures in four samples were determined along the Pd–In50Sn50 section using DTA. Liquidus temperatures were used in determination of parameters of model of melt, and temperature of other phase transitions to confirm the correctness of calculations.

The CALPHAD calculation of the In–Pd–Sn ternary was performed. Good agreement between the calculated and experimental data was reached, both for the data obtained in the present work and for the published data for phase equilibria and for thermodynamic properties of the melt. In addition, good agreement of the calculated phase transition temperatures with experimental DTA data not used in the optimization confirms that the obtained description of the In–Pd–Sn system is correct.

## Figures and Tables

**Table 1:** Solid phases: designations and crystal structures.

Phase	Prototype	Space Group	Phase Designation in Thermo-Calc^®^ Databases	References
In–Pd system				
(Pd)	Cu	Fm$\bar{3}$m	FCC_A1	[7]
αInPd_3_	Al_3_Zr	I4/mmm	αInPd_3_	[8]
βInPd_3_	Al_3_Ti	I4/mmm	βInPd_3_	[8]
αInPd_2_	Co_2_Si	Pnma	Pd_2_(In*_x_*Sn_1−*x*_)	[9]
βInPd_2_	– *	–	βInPd_2_	[7]
In_3_Pd_5_	Rh_5_Ge_3_	Pbam	In_3_Pd_5_	[9]
InPd	CsCl	Pm$\bar{3}$m	BCC_B2	[7]
In_3_Pd_2_	Ni_2_Al_3_	P$\bar{3}$m1	In_3_Pd_2_	[7]
In_7_Pd_3_	Ge_7_Ir_3_	Im$\bar{3}$m	In_7_Pd_3_	[7]
Pd–Sn system				
(Pd)	Cu	Fm$\bar{3}$m	FCC_A1	[10]
Pd_3_Sn	AuCu_3_	Pm$\bar{3}$m	Pd_3_Sn	[10]
Pd_2_Sn	Co_2_Si	Pnma	Pd_2_(In*_x_*Sn_1−*x*_)	[10]
γ–Pd_2−x_Sn	Ni_2_In	P6_3_/mmc	γ–Pd_2–x_Sn	[10]
Pd_20_Sn_13_	Pd_13_Sn_9_	P3_2_21	Pd_20_Sn_13_	[10]
PdSn	FeAs	Pnma	PdSn	[10]
Pd_5_Sn_7_	Pd_5_Sn_7_	C2/m	Pd_5_Sn_7_	[10]
PdSn_2_	PdSn_2_	Aba2	PdSn_2_	[10]
PdSn_3_	PdSn_3_	Cmca	PdSn_3_	[10]
PdSn_4_	PdSn_4_	Aba2	PdSn_4_	[10]
αPd_3_Sn_2_	–	–	αPd_3_Sn_2_	[10]
βPd_3_Sn_2_	–	–	βPd_3_Sn_2_	[10]
δPd_3_Sn_2_	–	–	δPd_3_Sn_2_	[10]
In–Sn system				
(In)	In	I4/mmm	TETRAGONAL_A6	[11]
β	In	I4/mmm	TET_ALPHA1	[11]
γ	Hg_0.1_Sn_0.9_	P6/mmm	INSN_GAMMA	[11]
(Sn)	βSn	I4_1_/amd	BCT_A5	[11]

* Structure not established due to rapid martensitic transitions during quenching.

**Table 2:** Experimental results of phase analysis in In–Pd–Sn system at 800 °C.

No.	Alloy Composition, at.%			Phase	Type	Phase Composition, at.%			Cell Parameters, Å		
	In	Pd	Sn			In	Pd	Sn	*a*	*b*	*c*
1	15.5	80.2	4.3	α	Cu	12.7	83.7	3.6	3.9452(8)	-	-
				InPd_3_	Al_3_Zr	17.0	77.5	5.5	4.04277(10)	-	15.3234(5)
				τ_1_	Al_3_Ti	-	-	-	4.0897(10)	-	7.481(3)
2	9.4	81.1	9.5	α	Cu	7.9	83.7	8.4	3.9427(7)	-	-
				τ_1_	Al_3_Ti	11.2	78.7	10.1	4.0835(6)	-	7.4757(14)
3	5.3	81.0	13.7	α	Cu	3.4	84.1	12.4	3.9413(8)	-	-
				τ_1_	Al_3_Ti	6.1	79.2	14.8	4.0740(9)	-	7.4784(23)
4	20.0	75.3	4.7	τ_1_	Al_3_Ti	20.0	75.3	4.7	4.1135(10)	-	7.4364(20)
5	14.9	76.3	8.8	τ_1_	Al_3_Ti	14.9	76.3	8.8	4.1053(8)	-	7.4634(18)
6	10.4	76.1	13.5	Pd_3_Sn	AuCu_3_	7.5	76.1	16.4	3.9943(4)	-	-
				τ_1_	Al_3_Ti	10.7	76.3	13.0	4.1001(10)	-	7.4806(20)
7	5.0	76.7	18.4	Pd_3_Sn	AuCu_3_	5.0	76.7	18.4	3.9864(6)	-	-
8	2	81	17	α	Cu	2.0	84.6	13.4	3.9416(6)	-	-
				Pd_3_Sn	AuCu_3_	3.1	76.2	20.7	3.9799(13)	-	-
				τ_1_	Al_3_Ti	–	–	–	4.0685(5)	-	7.5270(14)
9	3	78.5	18.5	τ_1_	Al_3_Ti	3.3	80.2	16.5	4.0692(5)	-	7.5178(14)
				Pd_3_Sn	AuCu_3_	3.6	76.6	19.8	3.9827(7)	-	-
				α	Cu	2.3	84.0	13.7	3.9409(8)	-	-
10	22.5	73.3	4.1	τ_1_	Al_3_Ti	19.6	75.7	4.7	4.1144(10)	-	7.434(3)
				Pd_2_(In*_x_*Sn_1−*x*_)	Co_2_Si	28.2	68.3	3.5	5.621(4)	4.2320(16)	8.201(4)
11	15.0	74.2	10.8	τ_1_	Al_3_Ti	12.3	77.0	10.8	4.1005(9)	-	7.4728(20)
				Pd_2_(In*_x_*Sn_1−*x*_)	Co_2_Si	21.1	67.7	11.2	5.6182(9)	4.2387(5)	8.2009(12)
				Pd_3_Sn	AuCu_3_	–	–	–	3.9977(9)	-	-
12	5.1	73.9	21.0	Pd_3_Sn	AuCu_3_	1.6	76.6	21.8	3.9815(4)		
				Pd_2_(In*_x_*Sn_1−*x*_)	Co_2_Si	10.4	68.9	20.8	5.6381(20)	4.2553(13)	8.173(10)
13	25.6	72.7	1.7	InPd_3_	Al_3_Zr	22.9	75.5	1.6	4.0724(16)	-	15.180(7)
				Pd_2_(In*_x_*Sn_1−*x*_)	Co_2_Si	31.1	67.4	1.6	5.6099(21)	4.2284(15)	8.213(4)
14	2.5	74.3	23.2	Pd_3_Sn	AuCu_3_	0.4	77.2	22.4	3.9784(3)	-	-

**Table 3:** Experimental results of phase analysis in In–Pd–Sn system at 500 °C.

No.	Alloy Composition, at.%			Phase	Type	Phase Composition, at.%			Cell Parameters, Å		
	In	Pd	Sn			In	Pd	Sn	*a*	*b*	*c*
1	14.6	80.4	5.1	α	Cu	14.3	82.1	3.7	3.9519(13)	-	-
				τ_1_	Al_3_Ti	17.0	78.1	4.9	4.0866(9)	-	7.483(2)
2	9.7	80.8	9.6	α	Cu	10.4	81.8	7.8	3.9543(18)	-	-
				τ_1_	Al_3_Ti	10.5	78.9	10.6	4.0830(6)	-	7.4866(20)
3	4.4	82.0	13.6	α	Cu	4.5	83.9	11.6	3.9546(13)	-	-
				τ_1_	Al_3_Ti	5.0	79.0	16.0	4.0700(10)	-	7.526(3)
4	20.4	75.1	4.5	τ_1_	Al_3_Ti	20.4	75.1	4.5	4.0960(11)	-	7.496(3)
5	15.5	75.2	9.3	τ_1_	Al_3_Ti	15.5	75.2	9.3	4.1018(11)	-	7.466(4)
6	10.4	76.3	13.3	τ_1_	Al_3_Ti	10.4	76.3	13.3	4.0924(5)	-	7.4872(18)
7	4.8	76.9	18.3	Pd_3_Sn	AuCu_3_	4.8	76.9	18.3	3.9845(5)	-	-
8	2.0	81.0	16.0	Pd_3_Sn	AuCu_3_	2.5	76.2	21.3	3.9760(16)	-	-
				τ_1_	Al_3_Ti	2.65	79.4	18.0	4.0735(8)	-	7.530(3)
				α	Cu	2.1	84.2	13.7	3.9445(14)	-	-
9	3.0	78.5	18.5	Pd_3_Sn	AuCu_3_	2.8	76.0	21.8	3.98387(11)	-	-
				τ_1_	Al_3_Ti	3.2	79.5	17.3	4.0711(17)	-	7.536(4)
				α	Cu	2.4	82.6	15.0	3.9352(17)	-	-
10	22.5	72.7	4.8	Pd_2_(In*_x_*Sn_1−*x*_)	Co_2_Si	27.6	68.1	4.3	5.6156(15)	4.2225(10)	8.2292(20)
				τ_1_	Al_3_Ti	20.8	74.3	4.9	4.1044(13)	-	7.460(3)
11	14.7	73.9	11.4	Pd_2_(In*_x_*Sn_1−*x*_)	Co_2_Si	21.8	66.8	11.4	5.6218(12)	4.2328(14)	8.206(12)
				τ_1_	Al_3_Ti	13.4	76.2	10.4	4.0987(14)	-	7.464(4)
12	5.6	73.6	20.8	Pd_2_(In*_x_*Sn_1−*x*_)	Co_2_Si	10.4	68.8	20.8	5.6411(15)	4.2583(8)	8.1521(23)
				Pd_3_Sn	AuCu_3_	2.1	77.2	20.7	3.9807(3)	-	-
13	26.9	71.6	1.5	Pd_2_(In*_x_*Sn_1−*x*_)	Co_2_Si	31.0	67.9	1.1	5.6105(19)	4.2263(19)	8.2194(22)
				InPd_3_	Al_3_Zr	23.1	76.1	0.8	4.0671(8)	-	15.256(6)
14	2.5	73.7	23.8	Pd_2_(In*_x_*Sn_1−*x*_)	Co_2_Si	5.3	68.5	26.2	5.6432(14)	4.2843(16)	8.131(3)
				Pd_3_Sn	AuCu_3_	1.2	77.2	21.6	3.9798(5)	-	-
15	23.3	67.6	9.1	Pd_2_(In*_x_*Sn_1−*x*_)	Co_2_Si	23.3	67.6	9.1	5.6167(11)	4.2226(12)	8.211(4)
16	16.0	68.5	15.5	Pd_2_(In*_x_*Sn_1−*x*_)	Co_2_Si	16.0	68.5	15.5	5.634(3)	4.2405(19)	8.2028(23)
17	8.9	68.0	23.2	Pd_2_(In*_x_*Sn_1−*x*_)	Co_2_Si	8.9	68.0	23.2	5.651(3)	4.2579(18)	8.164(3)
18	5.3	67.6	27.1	Pd_2_(In*_x_*Sn_1−*x*_)	Co_2_Si	5.3	67.6	27.1	5.6448(12)	4.2796(14)	8.131(3)

**Table 4:** The EDX and DTA results of the In–Pd–Sn system sample.

No.	Phase Area	Alloy Composition According to EDX, at.%			Temperature, °C	
		Pd	In	Sn	Liquidus	Phase Transitions
2.	α + τ_1_	80.8	9.7	9.6	1342	1333
6	τ_1_	76.3	10.4	13.3	1378	619 1055 1335
16	Pd_2_(In_x_Sn_1−x_)	68.5	16.0	15.5	1308	1094 1269
19	Pd_2_(In_x_Sn_1−x_) + Pd_20_Sn_13_	62.4	18.9	18.7	1278	990 1058 1213

**Table 5:** Experimental and calculated invariant reactions in the In–Pd–Sn system.

Invariant Reactions	Temperature, °C		
	Experiment [30]	Calculated [13]	Calculated, This Work
Liquid = βInPd_2_ + βInPd_3_	1303	1312	1305
βInPd_2_ + βInPd_3_ = αInPd_2_	1076	1076	1079
βInPd_3_ = αInPd_2_ + αInPd_3_	1059	1029	1058
Liquid = βInPd_3_	1365	1372	1365
Liquid = βInPd_3_ + α	1357	1350	1351
βInPd_3_ + α = αInPd_3_ βInPd_3_ = α + αInPd_3_	1223	1224	
			1206

**Table 6:** Thermodynamic parameters of phase models in the In–Pd–Sn system.

Phase	Model	Parameter (J/mol)	References
LIQUID	(In, Pd, Sn)_1_	^0^$L_{In:Pd}$ = −221,079 + 72.484∙T	[13]
		^1^$L_{In:Pd}$ = +105,789 − 42.023∙T	[13]
		^2^$L_{In:Pd}$ = −10,272+ 9.982∙T	[13]
		^0^$L_{In:Sn}$ = −711 − 1.6934∙T	[15]
		^1^$L_{In:Sn}$ = −64 − 1.3592∙T	[15]
		^0^$L_{Pd:Sn}$ = −218,959.83 + 50.86∙T	[19]
		^1^$L_{Pd:Sn}$ = −132,369.48 + 33.63∙T	[19]
		^2^$L_{Pd:Sn}$ = −2810.63 − 0.79∙T	[19]
		^3^$L_{Pd:Sn}$ = +29,608.28	[19]
		^0^$L_{In,Pd,Sn}^{}$ = +202,182 − 58.24∙T	This work, optimized
		^1^$L_{In,Pd,Sn}^{}$ = +275,610 − 94.07∙T	This work, optimized
		^2^$L_{In,Pd,Sn}^{}$ = +193,130 − 65.45∙T	This work, optimized
FCC_A1 (α)	(In, Pd, Sn)_1_(Va)_1_	^0^$L_{In,Pd:Va}$ = −209,569 + 64.241∙T	[13]
		^1^$L_{In,Pd:Va}$ = +108,049 − 44.206∙T	[13]
		^0^$L_{In,Sn:Va}$ = +2500 + 10∙T	This work, estimated
		^0^$L_{Pd,Sn:Va}$ = −115,000.00 + 57.68∙T	[19]
		^1^$L_{Pd,Sn:Va}$ = –441,623.62 + 36.16∙T	[19]
		^2^$L_{Pd,Sn:Va}$ = +223,668.60	[19]
		^0^$L_{In,Pd,Sn:Va}$ = +130,000	This work, estimated
BCC_B2 (InPd)	(In, Pd, Sn)_0.5_(Pd, Va)_0.5_	$G_{In:Pd}$ = –70918 + 116.068∙T − 21.968∙T∙ln(T) − 0.00304425∙T^2^	[13]
		$G_{Pd:Pd}$ = +GBCCPD	[13]
		$G_{In:Va}$ = +5000 − 0.5∙T + 0.5∙GBCCIN	[13]
		$G_{Pd:Va}$ = $G_{In:Va}$ + $G_{Pd:Pd}-G_{In:Pd}$	[13]
		$G_{Sn:Pd}$ = −43,000 + 0.5∙GHSERSN+ 0.5∙GHSERPD	This work, estimated
		$G_{Sn:Va}$ = +5000 + 0.5∙GBCCSN	This work, estimated
		^0^$L_{In,Pd:Pd}$ = −79,973 + 22.962∙T	[13]
		^1^$L_{In,Pd:Pd}$ = +12,078	[13]
		^0^$L_{In,Pd:Va}$ = −79,973 + 22.962∙T	[13]
		^1^$L_{In,Pd:Va}$ = +12,078	[13]
		^0^$L_{In:Pd,Va}$ = −1918	[13]
		^0^$L_{Pd:Pd,Va}$ = −1918	[13]
		^0^$L_{In,Sn:Pd}$ = −1500	This work, estimated
BCT_A5	(In, Sn)_1_	^0^$L_{In,Sn}$ = −5297 + 10.3∙T	This work, optimized
TETRAGONAL_A6	(In, Sn)_1_	^0^$L_{In,Sn}$ = +743 − 3.3139∙T	[15]
		^1^$L_{In,Sn}$ = −1487	[15]
TET_ALPHA1 (β)	(In, Sn)_1_	^0^$L_{In,Sn}$ = −1003–2.4∙T	This work, optimized
		^1^$L_{In,Sn}$ = −406 + 0.479∙T	This work, optimized
In_7_Pd_3_	(In, Sn)_0.71_(Pd)_0.29_	$G_{In:Pd}$ = −48,676 + 126.534∙T − 25.3376∙T∙ln(T)	[13]
		$G_{Sn:Pd}$ = −30,000 + 0.71∙GHSERSN +0.29∙GHSERPD	This work, estimated
In_3_Pd_2_	(In)_0.6_(Pd)_0.4_	$G_{In:Pd}$ = −63,165 + 109.678∙T − 0.00436412∙T^2^ − 21.1993∙T∙ln(T)	[13]
In_3_Pd_5_	(In)_0.375_(Pd)_0.625_	$G_{In:Pd}$ = −67,420 + 118.289∙T − 0.00328628∙T_2_ − 22.1055∙T∙ln(T)	[13]
βInPd_2_	(In)_0.34_(Pd)_0.66_	$G_{In:Pd}$ = −58,730 + 13.885∙T + 0.34∙GHSERIN + 0.66∙GHSERPD	[13]
βInPd_3_ (τ_1_)	(In, Pd, Sn)_0.26_(Pd)_0.74_	$G_{In:Pd}$ = −53,225.4 + 13.153∙T + 0.26∙GHSERIN + 0.74∙GHSERPD	This work, optimized
		$G_{Pd:Pd}$ = +425 + GHSERPD	This work, estimated
		$G_{Sn:Pd}$ = −63,000 + 172.71∙T–30.2744878∙T∙ln(T) + 131,596.913∙T^−1^ + 0.0023214421∙T^2^ − 9.236∙10^−7^∙T^3^	This work, estimated
		^0^$L_{In,Pd:Pd}$ = −1387.7	This work, optimized
		^0^$L_{In,Sn:Pd}$ = −17,450 + 5.75∙T	This work, estimated
		^1^$L_{In,Sn:Pd}$ = +5000	This work, estimated
		^2^$L_{In,Sn:Pd}$ = −5058 − 3.1∙T	This work, estimated
		^0^$L_{Pd,Sn:Pd}$ = −28,996 + 6.9∙T	This work, estimated
		^1^$L_{Pd,Sn:Pd}$ = +24,000	This work, estimated
αInPd_3_	(In, Sn)_0.25_(Pd)_0.75_	$G_{In:Pd}$ = –54,212 + 14.320∙T + 0.25∙GHSERIN + 0.75∙GHSERPD	[13]
		$G_{Sn:Pd}$ = −65,000 + 172.71∙T–30.2744878∙T∙ln(T) + 131,596.913∙T^−1^ + 0.0023214421∙T^2^ − 9.236∙10^−7^∙T^3^	This work, estimated
Pd_3_Sn	(In, Pd, Sn)_0.75_(In, Pd, Sn)_0.25_	$G_{In:In}$ = +5000 + GHSERIN	This work, estimated
		$G_{In:Pd}$ = +5000 + 0.75∙GHSERIN + 0.25∙GHSERPD	This work, estimated
		$G_{In:Sn}$ = +0.75∙GHSERIN + 0.25∙GHSERSN	This work, estimated
		$G_{Pd:In}$ = −53,712 + 14.320∙T + 0.25∙GHSERIN + 0.75∙GHSERPD	This work, estimated
		$G_{Pd:Pd}$ = +5000 + GHSERPD	[19]
		$G_{Pd:Sn}$ = −67,551.69 + 172.71∙T − 30.2744878∙T∙ln(T) + 131596.913∙T^−1^ + 0.0023214421∙T^2^ − 9.236∙10^−7^∙T^3^	[19]
		$G_{Sn:In}$ = +0.75∙GHSERSN + 0.25∙GHSERIN	[19]
		$G_{Sn:Pd}$ = +5000 + 0.75∙GHSERSN + 0.25∙GHSERPD	[19]
		$G_{Sn:Sn}$ = +5000 + GHSERSN	[19]
		^0^$L_{Pd:Pd,Sn}$ = −18,811.83 + 5.7∙T	[19]
Pd_2_(In*_x_*Sn_1–*x*_)	(Pd)_0.667_(In, Sn)_0.333_	$G_{Pd:In}$ = −65,890 + 121.070∙T − 0.00260306∙T^2^ − 22.5322∙T∙ln(T)	[13]
		$G_{Pd:Sn}$ = −72,964 + 162.34∙T − 29.4108924∙T∙ln(T) + 108,177.878∙T^−1^ + 0.0064877∙T^2^ − 2.163328∙10^−6^∙T^3^	This work, estimated
		^0^$L_{Pd:In,Sn}$ = –2500	This work, estimated
γ–Pd_2–x_Sn	(Pd)_1_(Sn)_1_(Pd, Va)_1_	$G_{Pd:Sn:Pd}^{}$ = −186,900.46 + 29.32∙T + 2∙GHSERPD + GHSERSN	[19]
		$G_{Pd:Sn:Va}^{}$ = −111,949.47 + 24.44∙T +GHSERPD + GHSERSN	[19]
		^0^$L_{Pd:Sn:Pd,Va}^{}$ = −11,461.63	[19]
		^1^$L_{Pd:Sn:Pd,Va}^{}$ = −21,904.12	[19]
Pd_20_Sn_13_	(In, Pd, Sn)_0.6_(In, Pd, Sn)_0.4_	$G_{In:Pd}$ = +5000 + 0.6∙GHSERIN + 0.4∙GHSERPD	This work, estimated
		$G_{Pd:In}$ = −62,800 + 18∙T + 0.6∙GHSERPD + 0.4∙GHSERIN	This work, estimated
		$G_{Pd:Pd}$ = +5000 + GHSERPD	[19]
		$G_{Pd:Sn}$ = −64,648.47 + 11.22∙T + 0.6∙GHSERPD + 0.4∙GHSERSN	[19]
		$G_{Sn:Pd}$ = +5000 + 0.6∙GHSERSN + 0.4∙GHSERPD	[19]
		$G_{Sn:Sn}$ = +5000 + GHSERSN	[19]
		^0^$L_{Pd:Pd,Sn}$ = −67,298.65 + 41.95∙T	[19]
		^1^$L_{Pd:Pd,Sn}$ = +10,657.92 + 21.09∙T	[19]
		^0^$L_{Pd,Sn:Sn}$ = −33,166.40 + 14.45∙T	[19]
PdSn	(Pd, Va)_0.5_(In, Pd, Sn)_0.5_	$G_{Pd:Pd}$ = +5000 + GHSERPD	[19]
		$G_{Va:Pd}$ = +8000 + 0.5∙GHSERPD	[19]
		$G_{Va:Sn}$ = +15,000 + 0.5∙GHSERSN	[19]
		$G_{Pd:Sn}$ = −68,723.03 + 147.77∙T − 27.1668054∙T∙ln(T) + 59,595.362∙T^−1^+ 0.00200199∙T^2^ − 1.3∙10^−6^∙T^3^	[19]
		$G_{Pd:In}$ = −65,325 + 25∙T + 0.5∙GHSERPD + 0.5∙GHSERIN	This work, estimated
		$G_{Va:In}$ = +5000 + 0.5∙GHSERIN	This work, estimated
		^0^$L_{Pd:Pd,Sn}$ = −45,236.19 + 1.84∙T	[19]
		^0^$L_{Pd,Va:Sn}$ = −11,203.70 + 3.84∙T	[19]
Pd_5_Sn_7_	(Pd)_5_(Sn)_7_	$G_{Pd:Sn}$ = −645,800 + 132.53∙T + 5∙GHSERPD + 7∙GHSERSN	[19]
PdSn_2_	(Pd, Sn)_1_(In, Sn)_2_	$G_{Pd:Sn}$ = −155,217.39 + 416.13∙T–80.5563671∙T∙ln(T) + 173,251.065∙T^−1^ + 0.00866748∙T^2^ − 4.5∙10^−6^∙T^3^	[19]
		$G_{Sn:Sn}$ = +15,000 + 3∙GHSERSN	[19]
		$G_{Pd:In}$ = −102,000 + GHSERPD + 2∙GHSERIN	This work, estimated
		$G_{Sn:In}$ = +5000 + GHSERSN + 2∙GHSERIN	This work, estimated
		^0^$L_{Pd,Sn:Sn}$ = −6001.20 + 5.25∙T	[19]
PdSn_3_	(Pd)_0.25_(Pd, Sn)_0.75_	$G_{Pd:Pd}$ = +6000 + GHSERPD	[19]
		$G_{Pd:Sn}$ = −42,780.00 + 143.67∙T − 27.528975∙T∙ln(T) + 57076.482∙T^−1^ + 0.00131302∙T^2^ − 1.5∙10^–6^∙T^3^	[19]
		^0^$L_{Pd:Pd,Sn}$ = −64,266.34 + 6.29∙T	[19]
		^1^$L_{Pd:Pd,Sn}$ = +54,238.32 − 6.29∙T	[19]
PdSn_4_	(Pd)_0.2_(Pd, Sn)_0.8_	$G_{Pd:Pd}$ = +5000 + GHSERPD	[19]
		$G_{Pd:Sn}$ = −35,467.77 + 106.19∙T − 21.371054∙T∙ln(T) + 10943.604∙T^−1^ − 0.00783969∙T^2^	[19]
		^0^$L_{Pd:Pd,Sn}$ = −61,437.17 + 7.44∙T	[19]
		^1^$L_{Pd:Pd,Sn}$ = +51,394.09 + 7.44∙T	[19]
αPd_3_Sn_2_	(Pd)_0.6_(Sn)_0.4_	$G_{\mathrm{Pd}:\mathrm{Sn}}$ = −64,735 + 11.99∙T + 0.6∙GHSERPD + 0.4∙GHSERSN	[19]
βPd_3_Sn_2_	(Pd)_0.6_(Sn)_0.4_	$G_{\mathrm{Pd}:\mathrm{Sn}}$ = −64,603.46 + 11.83∙T + 0.6∙GHSERPD + 0.4∙GHSERSN	[19]
δPd_3_Sn_2_	(Pd)_0.59_(Sn)_0.41_	$G_{\mathrm{Pd}:\mathrm{Sn}}$ = −64,196.15 + 11.05∙T + 0.59∙GHSERPD +0.41∙GHSERSN	[19]
INSN_GAMMA (γ)	(In, Sn)_1_	$G_{\mathrm{In}}$ = +10,292.5 − 7.64∙T + GHSERIN	[15]
		$G_{\mathrm{Sn}}$ = +925.3 − 1.7562∙T + GHSERSN	[15]
		$G_{\mathrm{In}:\mathrm{Sn}}$ = −15,715.5 + 19.3402∙T	[15]

## Data Availability

Not applicable.
